# Scleromalacia perforans: a case report

**DOI:** 10.1186/s13256-018-1686-z

**Published:** 2018-06-05

**Authors:** Muhammad Ishaq Ghauri, Syeda Urooj Riaz, Amir Husain, Asad Raza Jafri, Zara Tul Ain Bashir

**Affiliations:** 1 Department of Medicine, Jinnah Medical and Dental College, 22-23 Shaheed-e-Millat Road, Karachi, Pakistan; 2 Department of Ophthalmology, Jinnah Medical and Dental College, 22-23 Shaheed-e-Millat Road, Karachi, Pakistan; 3 Department of Ophthalmology, Karachi Medical and Dental College, Block M North Nazimabad, Karachi, Pakistan

**Keywords:** Rheumatoid arthritis, Scleromalacia perforans, Peripheral thinning of cornea, Ocular complication, Case report

## Abstract

**Background:**

Scleromalacia perforans is a rare ocular manifestation of rheumatoid arthritis which can potentially lead to blindness and is a late consequence in the course of the disease. It is an unusual finding for it to be present in a patient with joint pain without any rheumatologic progression of disease.

**Case presentation:**

We describe a rare case of scleromalacia perforans and orbital inflammatory disease in a 40-year-old Pakistani woman with apparently no associated rheumatologic deformity. It is rare in the sense that we usually see scleromalacia perforans with fixed deformities of rheumatoid arthritis in the hands or progressed systemic complications but not as a starting landmark of disease. She presented to us with pronounced eye manifestation which on further inquiry and investigation was found to be associated with rheumatoid arthritis. There was perforation of left globe on presentation and the right one was preserved. She visited various physicians and ophthalmologists and was treated with topical and systemic antibiotics but ended up losing sight in her left eye.

**Conclusion:**

We conclude that ocular manifestations, however rare they are, should be foreseen, investigated, and treated in patients with suspected arthritis as the complication is grave and sight threatening.

## Background

Rheumatoid arthritis (RA) is a systemic disease that can affect more than just the joints [1]. It is a disorder of autoimmune origin causing chronic inflammation [1]. A lthough inflammation of the tissue around the joints and inflammatory arthritis are characteristic features of RA, the disease causes inflammation not only in joints but also affects other organs of the body; hence, it is called rheumatoid disease [2].

Scleromalacia perforans is a rare form of anterior scleritis which readily presents as a blackish blue hue visible through a thin sclera [3]. No significant redness or pain is present but it is represented by progressive thinning of the sclera; it is a rare form of necrotizing anterior scleritis [4].

## Case presentation

A 40 year-old married Pakistani woman came to our out-patient department (OPD) on 19 July 2017 with pain and dryness in her left eye for 7 months, she also complained of progressive loss of vision in her left eye for 6 months. She complained of joint pains and stiffness for the past 2 months and she had pain with blurring of vision and photophobia in her right eye for the past few days.

She said that she had severe pain in both eyes with gritty sensation, around 8/10 on Visual Analog of Pain Scale (VAPS); she associated this pain with dryness of eyes and she stated that there had also been pus discharge from her left eye and progressive loss of vision. She visited various physicians in her local area and she was kept on antibiotics for 7 months but she had complete loss of vision by the time she presented to our ward.

She also complained of dry mouth, oral ulcers, and there was a history of stillbirth in eighth month of gestational amenorrhea 1 year back. She also complained of pain in all small joints of her hands associated with morning stiffness of 30–45 minutes. She had severe pain around 7/10 on VAPS associated with joint swelling. On examination she was vitally stable and anemic. The examination revealed acute synovitis in the joints of her hands and feet.

**Fig. 1:**
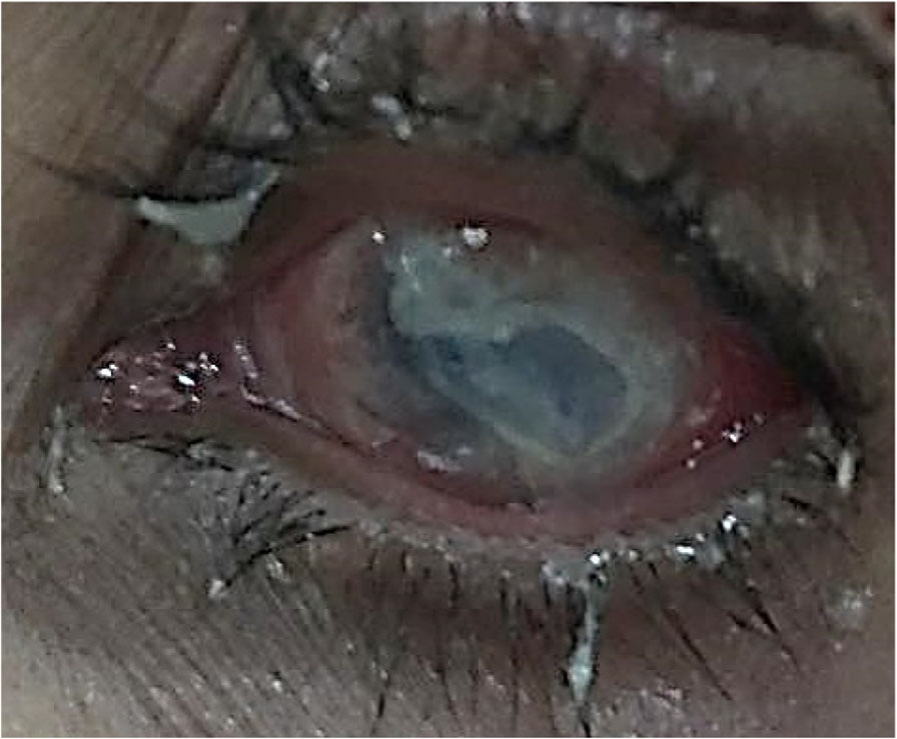
Left eye shows matted lashes, conjunctival injection, and opaque cornea with thinning at inferior half

**Fig. 2:**
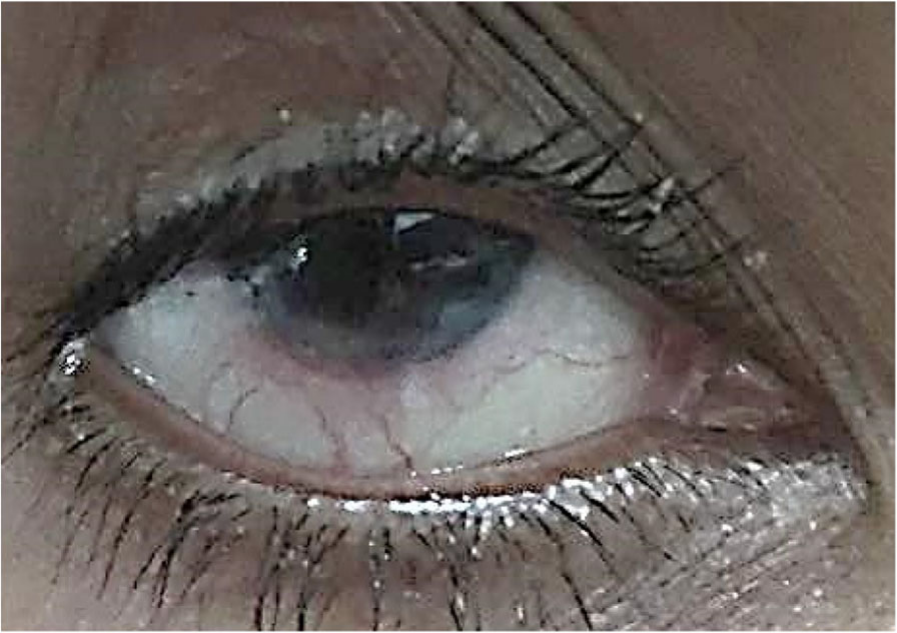
Right eye shows limbal injection at 3 o’clock to 7 o’clock corneal thinning at inferior half with exposure of uveal tissue

On examination she had no light perception in her left eye and her right eye was 6/6. A slit lamp examination of the cornea revealed a melted left cornea with red eye and her right eye had inferior peripheral ulceration (Figs. 1 and 2). There was scleral thinning in lower temporal quadrant with visible uveal tissue. There was peripheral corneal thinning in lower temporal quadrant.

On investigation she was anemic with hemoglobin of 10.2 mg/dl and a mean corpuscular volume (MCV) of 66. She had an erythrocyte sedimentation rate (ESR) of 15. She was found to have a strongly positive RA factor value of 191.9 IU/ml with a positive C-reactive protein (CRP) and anti-citrullinated cyclic polypeptide (CCP) of 32 IU/ml.

She was started on methylprednisolone at a dose of 1 g intravenously administered once a day in her right eye for 3 days, methotrexate 10 mg/week, analgesics, proton pump inhibitor, and folic acid supplementation to which she responded really well. The dose of steroid was tapered to 5 mg after 3 months and hydroxychloroquine 400 mg/day was added. She was asked to attend follow-up.

## Discussion

We reviewed more than 25 articles for discussion. None of the articles showed that such pronounced eye complications can precede joint disease. Lamba *et al.* discussed the different manifestations with percentages and emphasized the need for early detection and treatment [5]. RA is a systemic disease that can affect the eyes. The ophthalmic manifestations of RA include keratoconjunctivitis sicca, episcleritis, scleritis, peripheral ulcerative keratitis, and retinal vasculitis which are described in the literature [6].

Wu *et al*. discussed the presence of scleromalacia perforans in a patient with a history of joint pains for approximately 10 years [7]. This is in contrast to our case who developed ocular complications preceding joint disease.

Watson stated that scleritis is a severe inflammatory condition that is characterized by edema and inflammatory cell infiltration of the sclera often presenting as pain and redness [8]. It has a peak incidence in the fifth decade but is most common in the fourth to sixth decades [8]. McCluskey and Wakefield stated that in 40% of the cases it is bilateral [9]. As in our case, the patient lost her eye quickly; the goal of treatment in a patient with scleritis is to identify a potentially life-threatening systemic etiology, control ocular and systemic inflammation, make the patient comfortable, and prevent a scleral melt.

Watson and Hayreh divided scleritis into anterior and posterior types based on the anatomic distribution of disease [10]. Anterior scleritis is the most common type [11]; it includes necrotizing without inflammation (scleromalacia perforans) and necrotizing with inflammation, with diffuse and nodular as its types [10]. Zlatanović *et al*. stated that 27.2% of patients with RA presented with ocular complications [12].

In eyes with scleritis, a potentially blinding disease, the inflammatory process may extend to adjacent structures [13]. Okhravi *et al*. stated that anterior scleritis can be associated with multiple ocular manifestations [11, 13–15]. RA is associated with many extra-articular manifestations, which include ocular diseases.

The ocular manifestations must be addressed because of the high potential for permanent damage and blindness if they are allowed to run their course without intervention. Common complications of anterior scleritis often comprise peripheral corneal thinning, stromal keratitis, and peripheral ulcerative keratitis [15]. Posterior scleritis complications comprise exudative retinal detachment, optic disk edema, cystoid macular edema, and choroidal folds [11, 14]. In our case, our patient was treated initially as an infective etiology and lost one eye quickly during the course of the disease. Other common complications include scleral thinning and globe rupture with minor trauma [14].

Galor and Thorne stated that scleritis may be idiopathic or associated with local or systemic disease. Autoimmune conditions are found in approximately 40% of patients and infections in approximately 7% [14]. Our patient was treated with antibiotics by some ophthalmologists for 7 months.

Scleromalacia perforans, a form of autoimmune anterior scleritis, is a potential blinding disease which appears as a black area of scleral thinning surrounded by inflammatory tissue [16].

Although scleritis may be the initial sign of rheumatoid disease, it usually presents more than 10 years after the onset of arthritis but this was a contrasting feature in our case because she presented with advanced eye manifestation and no joint deformities. Multiple studies have found that patients with scleritis have more advanced joint disease and more extra-articular manifestations than do patients with RA without scleritis [15, 17–20].

Some studies showed an association of scleritis with other systemic manifestations and complications. Subcutaneous nodules appeared in 20–30% of patients with RA, their presence increased to approximately 50% in patients with scleritis [19]. It is often seen that pulmonary disorders are more common in patients with RA with scleritis than in patients who do not have scleritis. In addition, cardiac manifestations are more common in patients with RA who have a history of scleritis [17, 19, 21, 22]. Exacerbation of scleritis often occurs during a flare of RA [17, 18, 20, 23].

Fitzgerald discussed the development of osteoporosis in a patient with diagnosed RA and her response to a multidisciplinary approach [24]. Reddy *et al*. described bilateral corneal thinning in a patient with Wegener’s granulomatosis, which is a similar finding to ours as our patient has a bilateral disease [25].

Patients with scleritis have a higher morbidity and mortality rate [19, 21]. If left untreated with systemic medications, 36–45% of patients with scleritis and RA will die within 3 years of the onset of scleritis. This compares to a 3-year mortality rate of 18% in patients with RA without scleritis. Death is usually secondary to extra-articular vasculitis. Necrotizing scleritis is associated with a higher mortality than the other forms [17, 20, 23].

The most effective treatment of scleritis is aggressive and systemic. The use of non-steroidal anti-inflammatory drugs, corticosteroids, or immunomodulatory drugs is usually necessary in the treatment of scleritis [23]. Kahlenberg and Fox discussed the role of biological disease-modifying antirheumatic drugs (DMARDS) stating that they are a revolution in the treatment of RA [26]. Luwayi and Gurbaxani reported two cases of scleromalacia perforans treated with adalimumab with satisfactory results [27].

## Conclusions

RA is associated with many extra-articular manifestations, which include ocular diseases such as keratoconjunctivitis sicca, episcleritis, scleritis, peripheral ulcerative keratitis, and retinal vasculitis.

These concomitant ocular manifestations are of utmost concern and must be addressed because of the high potential for permanent damage and blindness if they are allowed to run their course without intervention. Collaborative efforts between the ophthalmologists and rheumatologists involved in the evaluation and treatment of patients with RA are essential to effectively manage any ocular complications that may arise.

## Abbreviations

CCP: Citrullinated cyclic polypeptide
CRP: C-reactive protein
DMARDS: Disease-modifying antirheumatic drugs
ESR: Erythrocyte sedimentation rate
MCV: Mean corpuscular volume
OPD: Out-patient department
RA: Rheumatoid arthritis
VAPS: Visual Analog of Pain Scale
